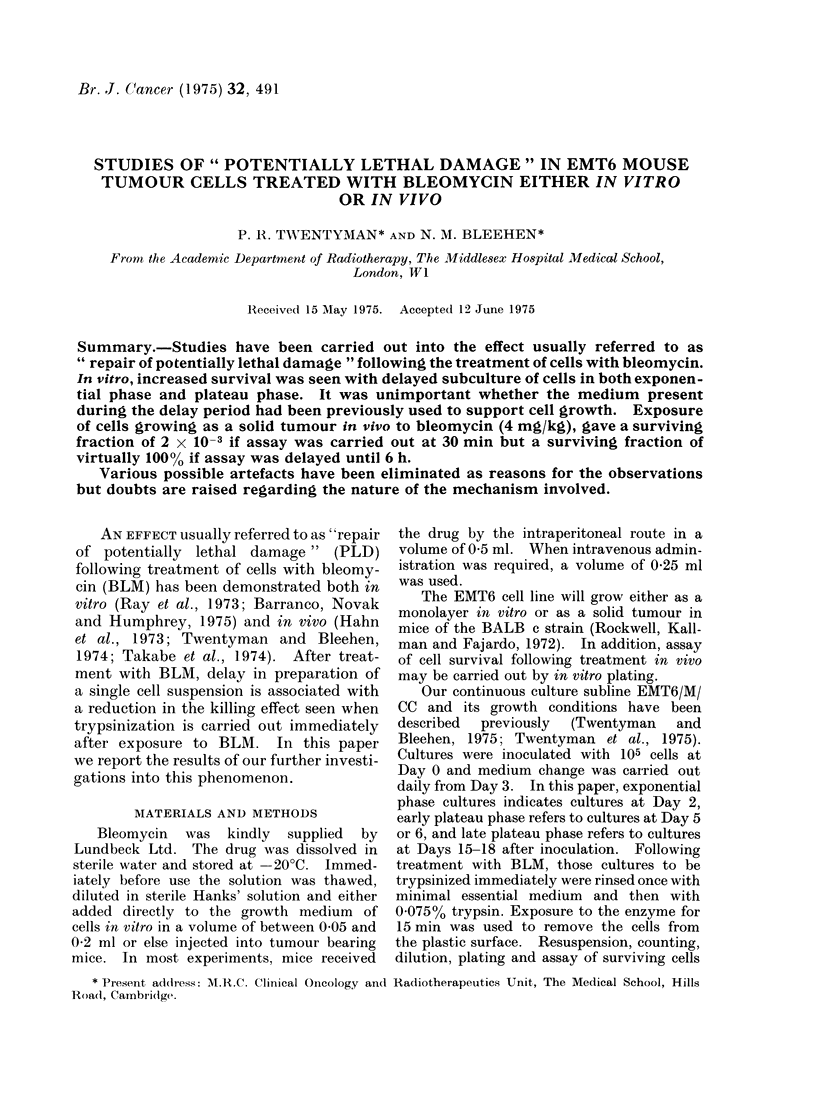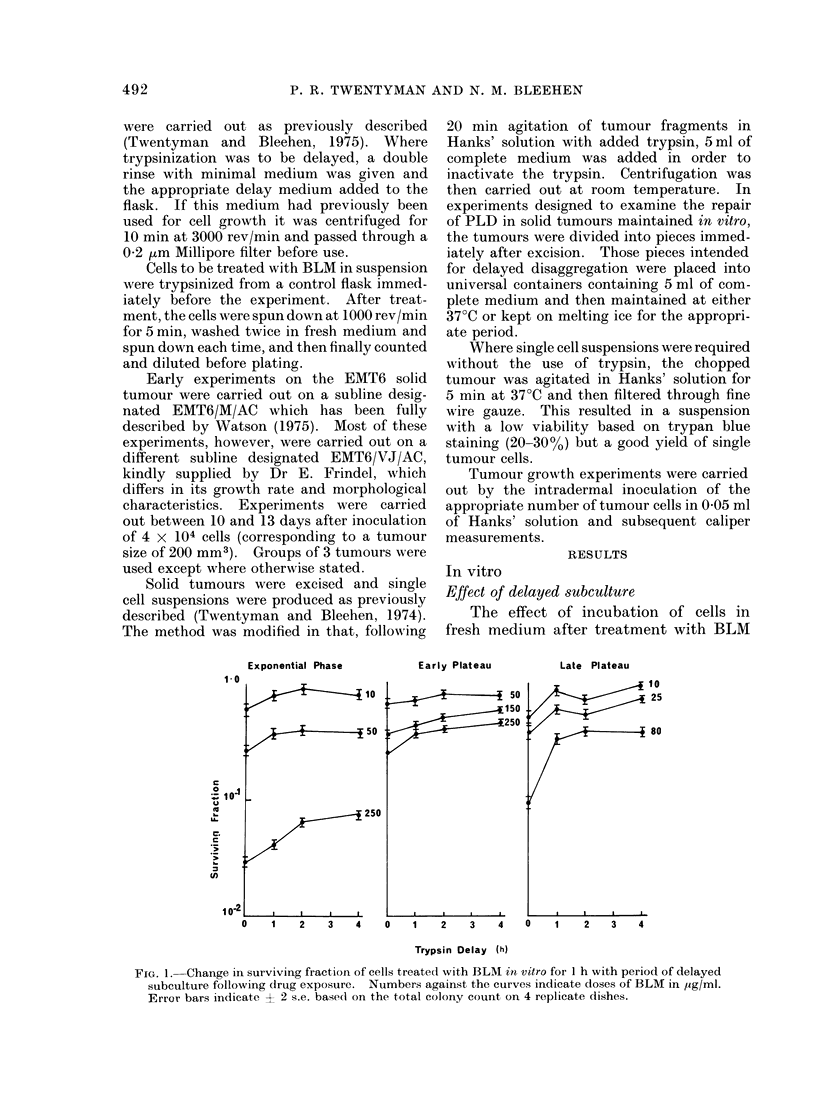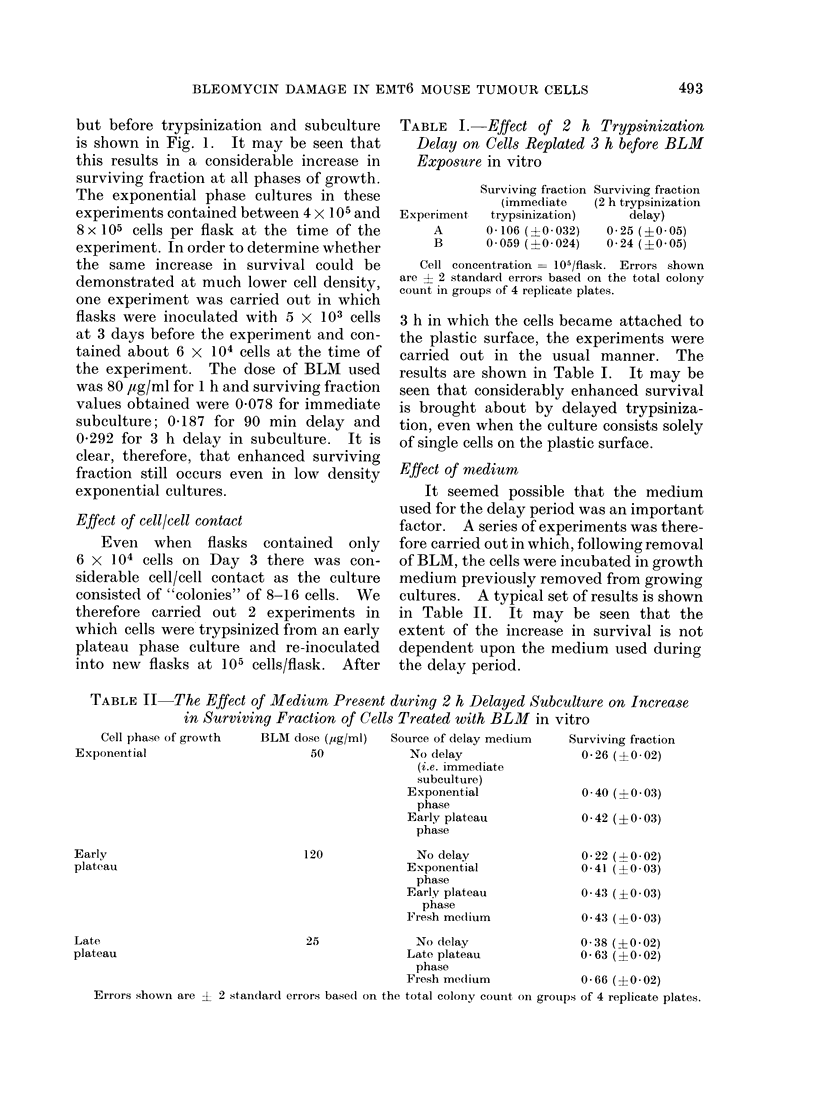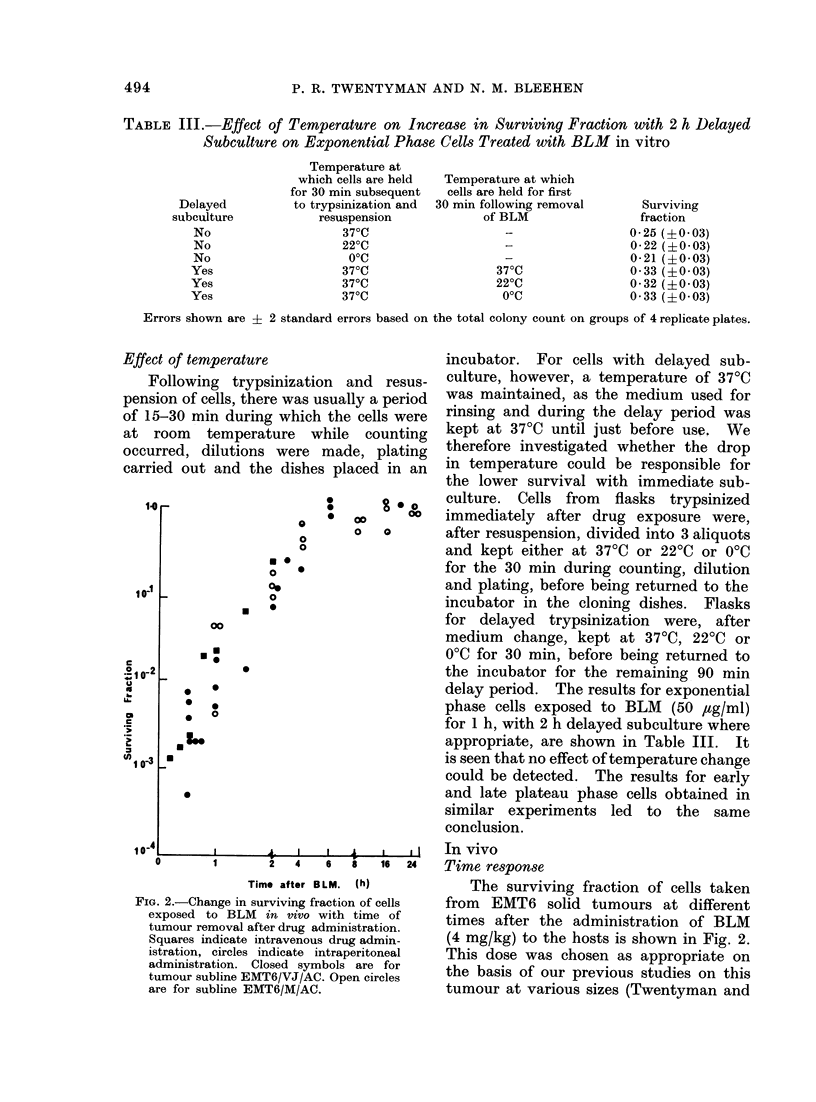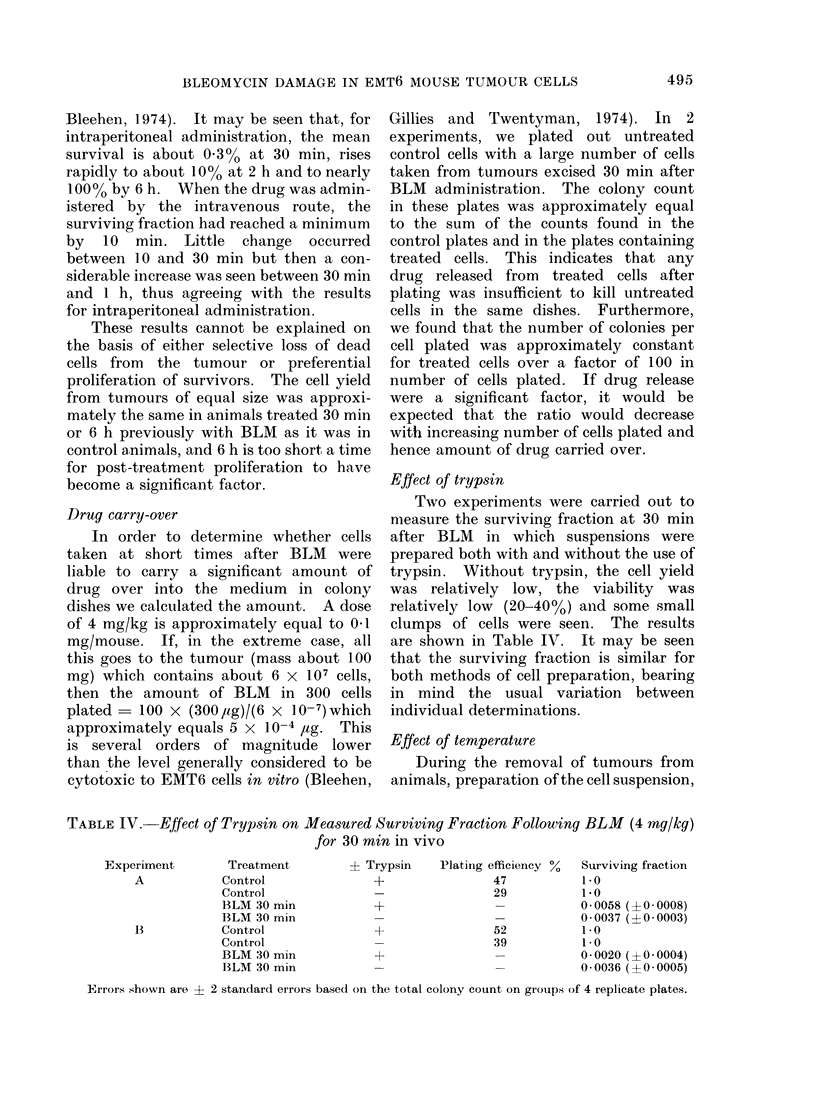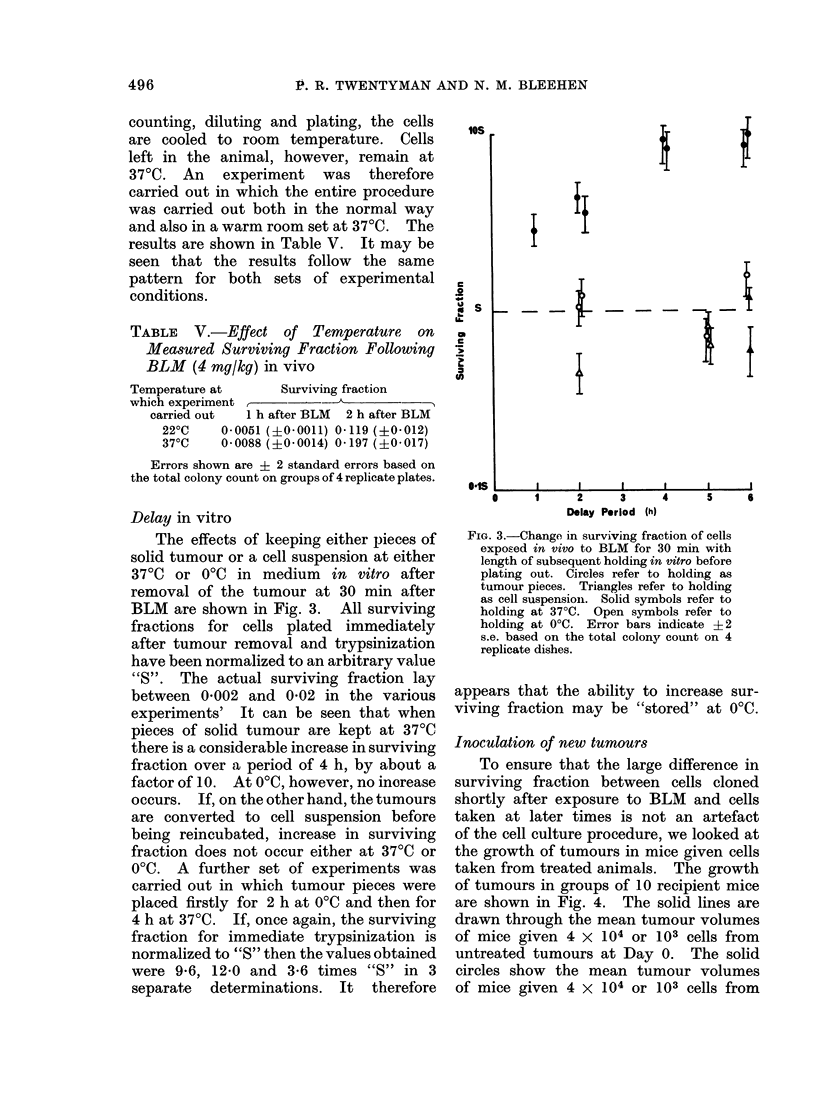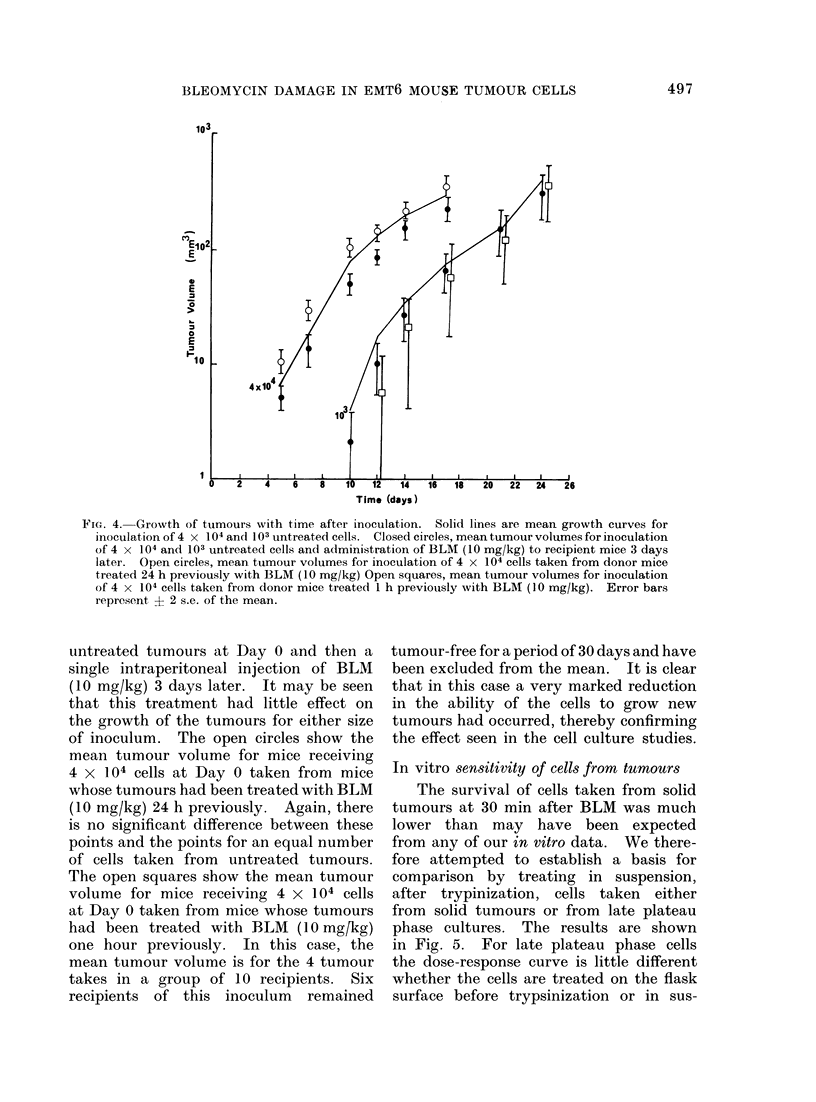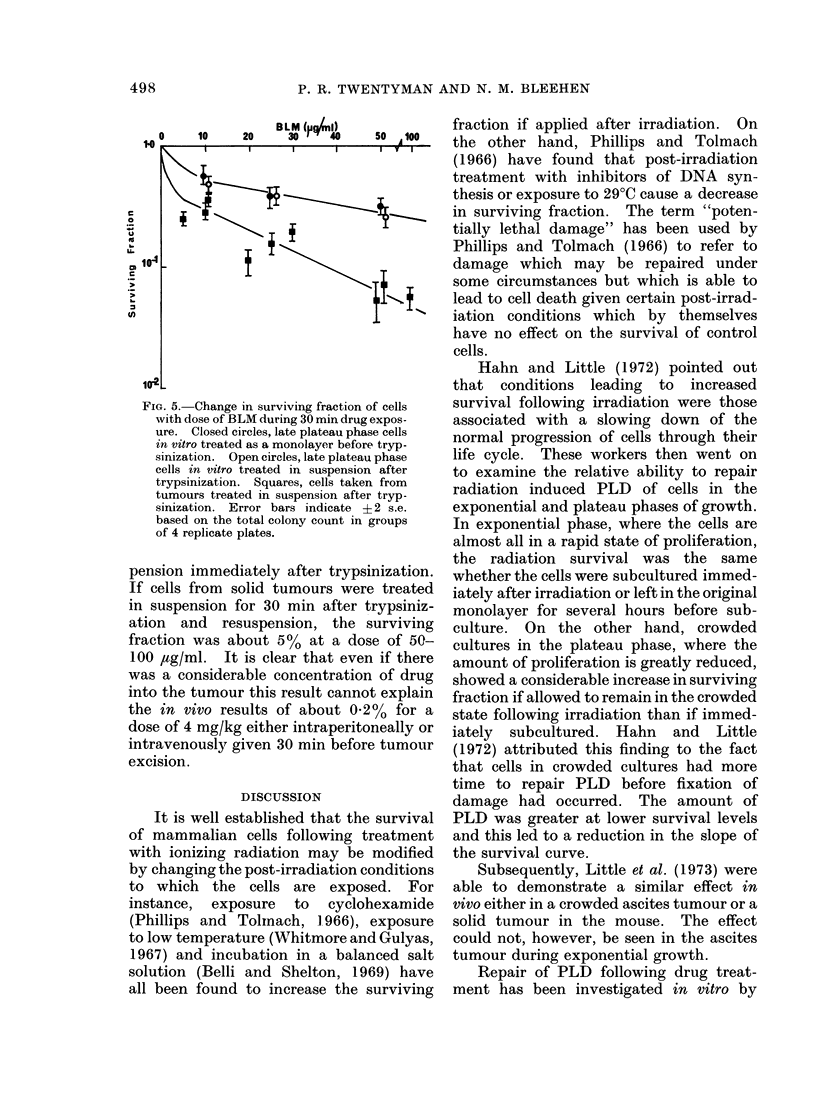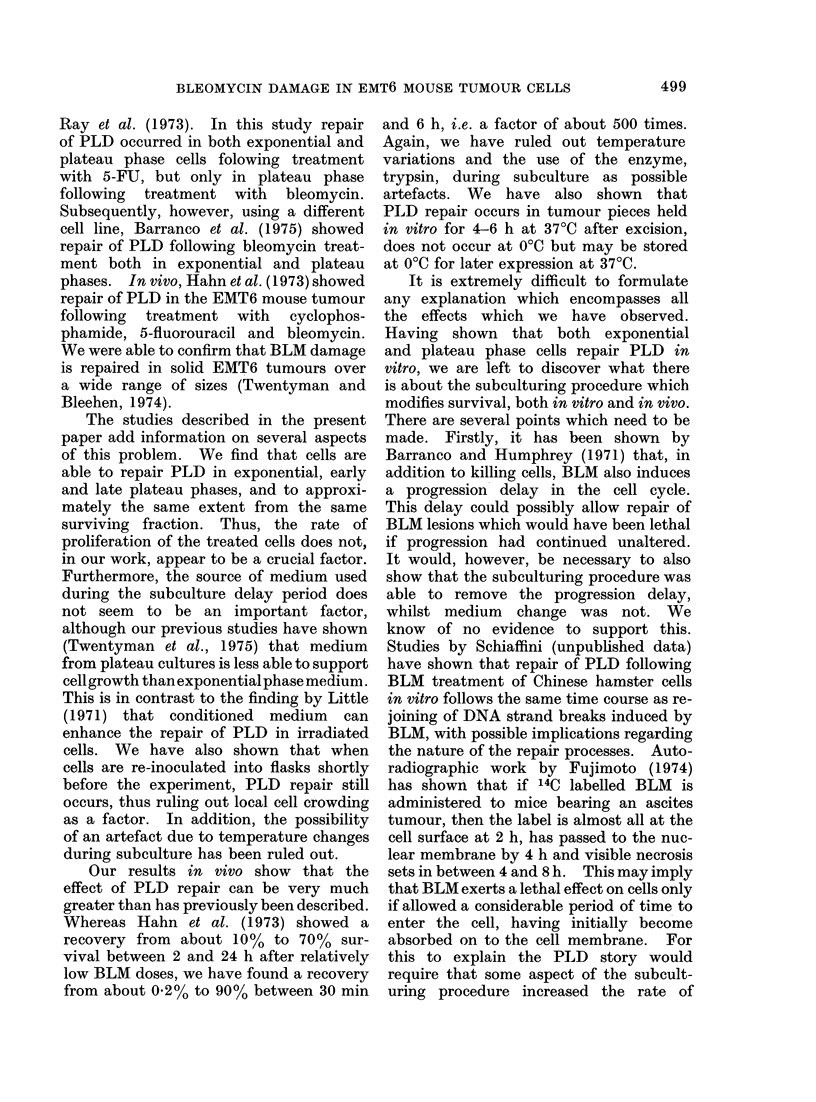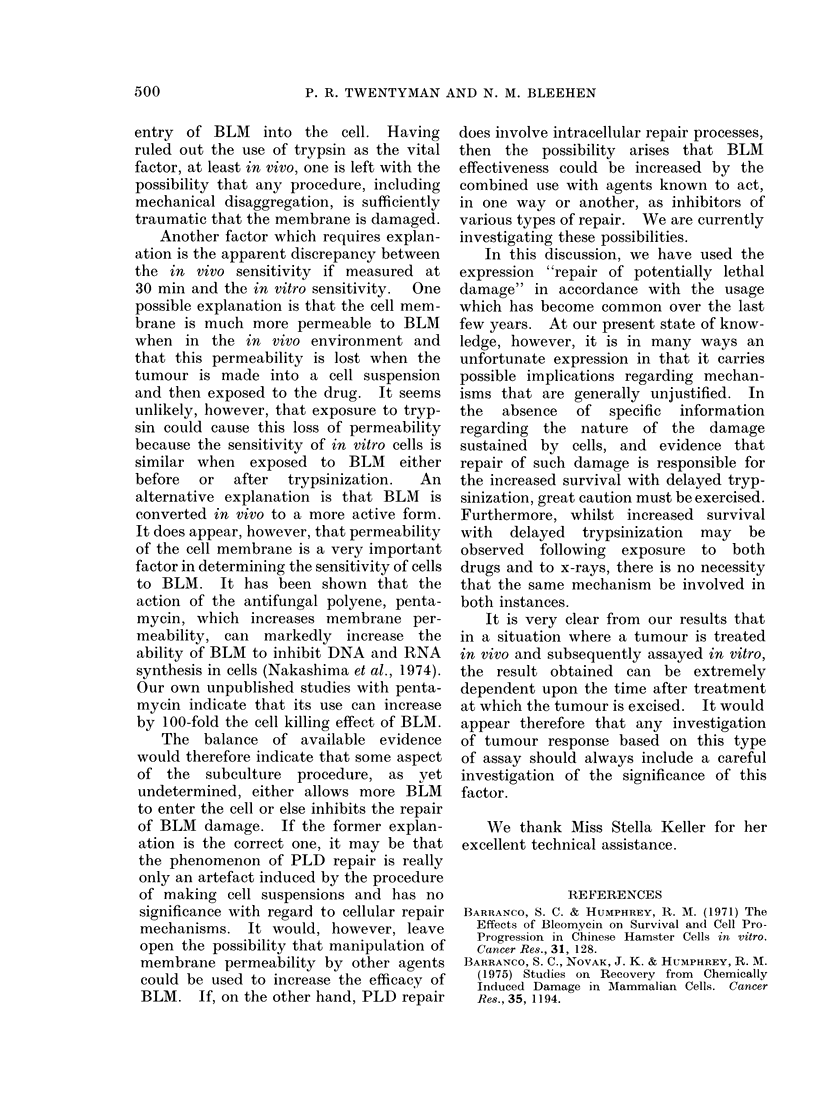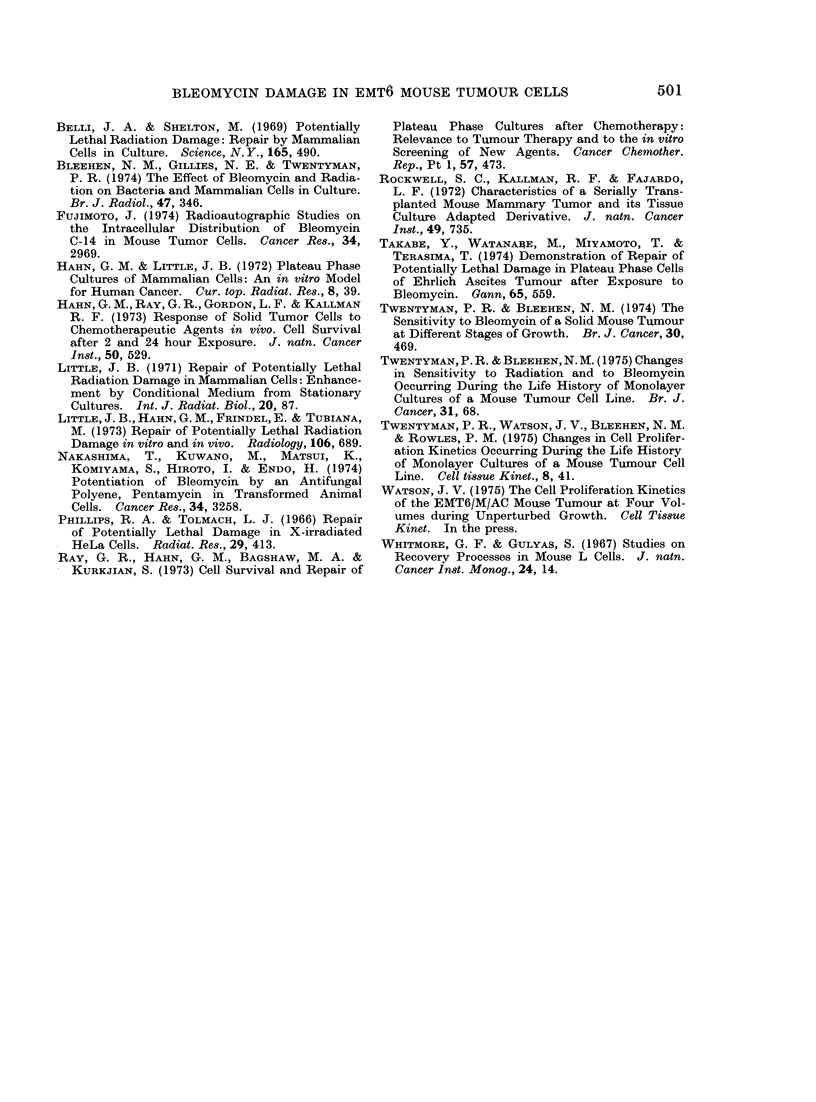# Studies of "potentially lethal damage" in EMT6 mouse tumour cells treated with bleomycin either in vitro or in vivo.

**DOI:** 10.1038/bjc.1975.251

**Published:** 1975-10

**Authors:** P. R. Twentyman, N. M. Bleehen

## Abstract

Studies have been carried out into the effect usually referred to as "repair to potentially lethal damage" following the treatment of cells with bleomycin. In vitro, increased survival was seen with delayed subculture of cells in both exponential phase and plateau phase. It was unimportant whether the medium present during the delay period had been previously used to support cell growth. Exposure of cells growing as a solid tumour in vivo to bleomycin (4 mg/kg), gave a surviving fraction of 2 X 10(-3) if assay was carried out at 30 min but a surviving fraction of virtually 100% if assay was delayed until 6 h. Various possible artefacts have been eliminated as reasons for the observations but doubts are raised regarding the nature of the mechanism involved.


*Br. J. Cancer* (1975) **32**, 491

### STUDIES OF " POTENTIALLY LETHAL DAMAGE " IN EMT6 MOUSE TUMOUR CELLS TREATED WITH BLEOMYCIN EITHER *IN VITRO* OR *IN VIVO*

P. R. TWENTYMAN* AND N. M. BLEEHEN*

*From the Academic Department of Radiotherapy, The Middlesex Hospital Medical School, London, W1*

Received 15 May 1975. Accepted 12 June 1975

**Summary.—Studies have been carried out into the effect usually referred to as " repair of potentially lethal damage " following the treatment of cells with bleomycin. *In vitro*, increased survival was seen with delayed subculture of cells in both exponential phase and plateau phase. It was unimportant whether the medium present during the delay period had been previously used to support cell growth. Exposure of cells growing as a solid tumour *in vivo* to bleomycin (4 mg/kg), gave a surviving fraction of $2 \times 10^{-3}$ if assay was carried out at 30 min but a surviving fraction of virtually 100% if assay was delayed until 6 h.**

**Various possible artefacts have been eliminated as reasons for the observations but doubts are raised regarding the nature of the mechanism involved.**

AN EFFECT usually referred to as "repair of potentially lethal damage" (PLD) following treatment of cells with bleomycin (BLM) has been demonstrated both *in vitro* (Ray *et al.*, 1973; Barranco, Novak and Humphrey, 1975) and *in vivo* (Hahn *et al.*, 1973; Twentyman and Bleehen, 1974; Takabe *et al.*, 1974). After treatment with BLM, delay in preparation of a single cell suspension is associated with a reduction in the killing effect seen when trypsinization is carried out immediately after exposure to BLM. In this paper we report the results of our further investigations into this phenomenon.

### MATERIALS AND METHODS

Bleomycin was kindly supplied by Lundbeck Ltd. The drug was dissolved in sterile water and stored at −20°C. Immediately before use the solution was thawed, diluted in sterile Hanks' solution and either added directly to the growth medium of cells *in vitro* in a volume of between 0·05 and 0·2 ml or else injected into tumour bearing mice. In most experiments, mice received the drug by the intraperitoneal route in a volume of 0·5 ml. When intravenous administration was required, a volume of 0·25 ml was used.

The EMT6 cell line will grow either as a monolayer *in vitro* or as a solid tumour in mice of the BALB c strain (Rockwell, Kallman and Fajardo, 1972). In addition, assay of cell survival following treatment *in vivo* may be carried out by *in vitro* plating.

Our continuous culture subline EMT6/M/CC and its growth conditions have been described previously (Twentyman and Bleehen, 1975; Twentyman *et al.*, 1975). Cultures were inoculated with $10^5$ cells at Day 0 and medium change was carried out daily from Day 3. In this paper, exponential phase cultures indicates cultures at Day 2, early plateau phase refers to cultures at Day 5 or 6, and late plateau phase refers to cultures at Days 15–18 after inoculation. Following treatment with BLM, those cultures to be trypsinized immediately were rinsed once with minimal essential medium and then with 0·075% trypsin. Exposure to the enzyme for 15 min was used to remove the cells from the plastic surface. Resuspension, counting, dilution, plating and assay of surviving cells

* Present address: M.R.C. Clinical Oncology and Radiotherapeutics Unit, The Medical School, Hills Road, Cambridge.

were carried out as previously described (Twentyman and Bleehen, 1975). Where trypsinization was to be delayed, a double rinse with minimal medium was given and the appropriate delay medium added to the flask. If this medium had previously been used for cell growth it was centrifuged for 10 min at 3000 rev/min and passed through a 0·2 μm Millipore filter before use.

Cells to be treated with BLM in suspension were trypsinized from a control flask immediately before the experiment. After treatment, the cells were spun down at 1000 rev/min for 5 min, washed twice in fresh medium and spun down each time, and then finally counted and diluted before plating.

Early experiments on the EMT6 solid tumour were carried out on a subline designated EMT6/M/AC which has been fully described by Watson (1975). Most of these experiments, however, were carried out on a different subline designated EMT6/VJ/AC, kindly supplied by Dr E. Frindel, which differs in its growth rate and morphological characteristics. Experiments were carried out between 10 and 13 days after inoculation of $4 \times 10^4$ cells (corresponding to a tumour size of 200 $mm^3$). Groups of 3 tumours were used except where otherwise stated.

Solid tumours were excised and single cell suspensions were produced as previously described (Twentyman and Bleehen, 1974). The method was modified in that, following 20 min agitation of tumour fragments in Hanks' solution with added trypsin, 5 ml of complete medium was added in order to inactivate the trypsin. Centrifugation was then carried out at room temperature. In experiments designed to examine the repair of PLD in solid tumours maintained *in vitro*, the tumours were divided into pieces immediately after excision. Those pieces intended for delayed disaggregation were placed into universal containers containing 5 ml of complete medium and then maintained at either 37°C or kept on melting ice for the appropriate period.

Where single cell suspensions were required without the use of trypsin, the chopped tumour was agitated in Hanks' solution for 5 min at 37°C and then filtered through fine wire gauze. This resulted in a suspension with a low viability based on trypan blue staining (20–30%) but a good yield of single tumour cells.

Tumour growth experiments were carried out by the intradermal inoculation of the appropriate number of tumour cells in 0·05 ml of Hanks' solution and subsequent caliper measurements.

### RESULTS

### In vitro

#### *Effect of delayed subculture*

The effect of incubation of cells in fresh medium after treatment with BLM

FIG. 1.—Change in surviving fraction of cells treated with BLM *in vitro* for 1 h with period of delayed subculture following drug exposure. Numbers against the curves indicate doses of BLM in μg/ml. Error bars indicate ± 2 s.e. based on the total colony count on 4 replicate dishes.

but before trypsinization and subculture is shown in Fig. 1. It may be seen that this results in a considerable increase in surviving fraction at all phases of growth. The exponential phase cultures in these experiments contained between $4 \times 10^5$ and $8 \times 10^5$ cells per flask at the time of the experiment. In order to determine whether the same increase in survival could be demonstrated at much lower cell density, one experiment was carried out in which flasks were inoculated with $5 \times 10^3$ cells at 3 days before the experiment and contained about $6 \times 10^4$ cells at the time of the experiment. The dose of BLM used was 80 μg/ml for 1 h and surviving fraction values obtained were 0·078 for immediate subculture; 0·187 for 90 min delay and 0·292 for 3 h delay in subculture. It is clear, therefore, that enhanced surviving fraction still occurs even in low density exponential cultures.

### *Effect of cell/cell contact*

Even when flasks contained only $6 \times 10^4$ cells on Day 3 there was considerable cell/cell contact as the culture consisted of "colonies" of 8–16 cells. We therefore carried out 2 experiments in which cells were trypsinized from an early plateau phase culture and re-inoculated into new flasks at $10^5$ cells/flask. After 3 h in which the cells became attached to the plastic surface, the experiments were carried out in the usual manner. The results are shown in Table I. It may be seen that considerably enhanced survival is brought about by delayed trypsinization, even when the culture consists solely of single cells on the plastic surface.

TABLE I.—*Effect of 2 h Trypsinization Delay on Cells Replated 3 h before BLM Exposure* in vitro

| Experiment | Surviving fraction (immediate trypsinization) | Surviving fraction (2 h trypsinization delay) |
|---|---|---|
| A | 0·106 (±0·032) | 0·25 (±0·05) |
| B | 0·059 (±0·024) | 0·24 (±0·05) |

Cell concentration = $10^5$/flask. Errors shown are ± 2 standard errors based on the total colony count in groups of 4 replicate plates.

### *Effect of medium*

It seemed possible that the medium used for the delay period was an important factor. A series of experiments was therefore carried out in which, following removal of BLM, the cells were incubated in growth medium previously removed from growing cultures. A typical set of results is shown in Table II. It may be seen that the extent of the increase in survival is not dependent upon the medium used during the delay period.

TABLE II—*The Effect of Medium Present during 2 h Delayed Subculture on Increase in Surviving Fraction of Cells Treated with BLM* in vitro

| Cell phase of growth | BLM dose (μg/ml) | Source of delay medium | Surviving fraction |
|---|---|---|---|
| Exponential | 50 | No delay (*i.e.* immediate subculture) | 0·26 (±0·02) |
| | | Exponential phase | 0·40 (±0·03) |
| | | Early plateau phase | 0·42 (±0·03) |
| Early plateau | 120 | No delay | 0·22 (±0·02) |
| | | Exponential phase | 0·41 (±0·03) |
| | | Early plateau phase | 0·43 (±0·03) |
| | | Fresh medium | 0·43 (±0·03) |
| Late plateau | 25 | No delay | 0·38 (±0·02) |
| | | Late plateau phase | 0·63 (±0·02) |
| | | Fresh medium | 0·66 (±0·02) |

Errors shown are ± 2 standard errors based on the total colony count on groups of 4 replicate plates.

TABLE III.—*Effect of Temperature on Increase in Surviving Fraction with 2 h Delayed Subculture on Exponential Phase Cells Treated with BLM* in vitro

| Delayed subculture | Temperature at which cells are held for 30 min subsequent to trypsinization and resuspension | Temperature at which cells are held for first 30 min following removal of BLM | Surviving fraction |
|---|---|---|---|
| No | 37°C | – | 0·25 (±0·03) |
| No | 22°C | – | 0·22 (±0·03) |
| No | 0°C | – | 0·21 (±0·03) |
| Yes | 37°C | 37°C | 0·33 (±0·03) |
| Yes | 37°C | 22°C | 0·32 (±0·03) |
| Yes | 37°C | 0°C | 0·33 (±0·03) |

Errors shown are ± 2 standard errors based on the total colony count on groups of 4 replicate plates.

*Effect of temperature*

Following trypsinization and resuspension of cells, there was usually a period of 15–30 min during which the cells were at room temperature while counting occurred, dilutions were made, plating carried out and the dishes placed in an incubator. For cells with delayed subculture, however, a temperature of 37°C was maintained, as the medium used for rinsing and during the delay period was kept at 37°C until just before use. We therefore investigated whether the drop in temperature could be responsible for the lower survival with immediate subculture. Cells from flasks trypsinized immediately after drug exposure were, after resuspension, divided into 3 aliquots and kept either at 37°C or 22°C or 0°C for the 30 min during counting, dilution and plating, before being returned to the incubator in the cloning dishes. Flasks for delayed trypsinization were, after medium change, kept at 37°C, 22°C or 0°C for 30 min, before being returned to the incubator for the remaining 90 min delay period. The results for exponential phase cells exposed to BLM (50 $\mu$g/ml) for 1 h, with 2 h delayed subculture where appropriate, are shown in Table III. It is seen that no effect of temperature change could be detected. The results for early and late plateau phase cells obtained in similar experiments led to the same conclusion.

FIG. 2.—Change in surviving fraction of cells exposed to BLM *in vivo* with time of tumour removal after drug administration. Squares indicate intravenous drug administration, circles indicate intraperitoneal administration. Closed symbols are for tumour subline EMT6/VJ/AC. Open circles are for subline EMT6/M/AC.

In vivo

*Time response*

The surviving fraction of cells taken from EMT6 solid tumours at different times after the administration of BLM (4 mg/kg) to the hosts is shown in Fig. 2. This dose was chosen as appropriate on the basis of our previous studies on this tumour at various sizes (Twentyman and

Bleehen, 1974). It may be seen that, for intraperitoneal administration, the mean survival is about 0·3% at 30 min, rises rapidly to about 10% at 2 h and to nearly 100% by 6 h. When the drug was administered by the intravenous route, the surviving fraction had reached a minimum by 10 min. Little change occurred between 10 and 30 min but then a considerable increase was seen between 30 min and 1 h, thus agreeing with the results for intraperitoneal administration.

These results cannot be explained on the basis of either selective loss of dead cells from the tumour or preferential proliferation of survivors. The cell yield from tumours of equal size was approximately the same in animals treated 30 min or 6 h previously with BLM as it was in control animals, and 6 h is too short a time for post-treatment proliferation to have become a significant factor.

*Drug carry-over*

In order to determine whether cells taken at short times after BLM were liable to carry a significant amount of drug over into the medium in colony dishes we calculated the amount. A dose of 4 mg/kg is approximately equal to 0·1 mg/mouse. If, in the extreme case, all this goes to the tumour (mass about 100 mg) which contains about $6 \times 10^7$ cells, then the amount of BLM in 300 cells plated $= 100 \times (300\,\mu g)/(6 \times 10^{-7})$ which approximately equals $5 \times 10^{-4}\,\mu g$. This is several orders of magnitude lower than the level generally considered to be cytotoxic to EMT6 cells *in vitro* (Bleehen, Gillies and Twentyman, 1974). In 2 experiments, we plated out untreated control cells with a large number of cells taken from tumours excised 30 min after BLM administration. The colony count in these plates was approximately equal to the sum of the counts found in the control plates and in the plates containing treated cells. This indicates that any drug released from treated cells after plating was insufficient to kill untreated cells in the same dishes. Furthermore, we found that the number of colonies per cell plated was approximately constant for treated cells over a factor of 100 in number of cells plated. If drug release were a significant factor, it would be expected that the ratio would decrease with increasing number of cells plated and hence amount of drug carried over.

*Effect of trypsin*

Two experiments were carried out to measure the surviving fraction at 30 min after BLM in which suspensions were prepared both with and without the use of trypsin. Without trypsin, the cell yield was relatively low, the viability was relatively low (20–40%) and some small clumps of cells were seen. The results are shown in Table IV. It may be seen that the surviving fraction is similar for both methods of cell preparation, bearing in mind the usual variation between individual determinations.

*Effect of temperature*

During the removal of tumours from animals, preparation of the cell suspension,

TABLE IV.—*Effect of Trypsin on Measured Surviving Fraction Following BLM* (4 *mg/kg*) *for* 30 *min* in vivo

| Experiment | Treatment | ± Trypsin | Plating efficiency % | Surviving fraction |
|---|---|---|---|---|
| A | Control | + | 47 | 1·0 |
| | Control | — | 29 | 1·0 |
| | BLM 30 min | + | — | 0·0058 (±0·0008) |
| | BLM 30 min | — | — | 0·0037 (±0·0003) |
| B | Control | + | 52 | 1·0 |
| | Control | — | 39 | 1·0 |
| | BLM 30 min | + | — | 0·0020 (±0·0004) |
| | BLM 30 min | — | — | 0·0036 (±0·0005) |

Errors shown are ± 2 standard errors based on the total colony count on groups of 4 replicate plates.

counting, diluting and plating, the cells are cooled to room temperature. Cells left in the animal, however, remain at 37°C. An experiment was therefore carried out in which the entire procedure was carried out both in the normal way and also in a warm room set at 37°C. The results are shown in Table V. It may be seen that the results follow the same pattern for both sets of experimental conditions.

TABLE V.—*Effect of Temperature on Measured Surviving Fraction Following BLM (4 mg/kg)* in vivo

| Temperature at which experiment carried out | Surviving fraction 1 h after BLM | 2 h after BLM |
|---|---|---|
| 22°C | 0·0051 (±0·0011) | 0·119 (±0·012) |
| 37°C | 0·0088 (±0·0014) | 0·197 (±0·017) |

Errors shown are ± 2 standard errors based on the total colony count on groups of 4 replicate plates.

*Delay* in vitro

The effects of keeping either pieces of solid tumour or a cell suspension at either 37°C or 0°C in medium *in vitro* after removal of the tumour at 30 min after BLM are shown in Fig. 3. All surviving fractions for cells plated immediately after tumour removal and trypsinization have been normalized to an arbitrary value "S". The actual surviving fraction lay between 0·002 and 0·02 in the various experiments' It can be seen that when pieces of solid tumour are kept at 37°C there is a considerable increase in surviving fraction over a period of 4 h, by about a factor of 10. At 0°C, however, no increase occurs. If, on the other hand, the tumours are converted to cell suspension before being reincubated, increase in surviving fraction does not occur either at 37°C or 0°C. A further set of experiments was carried out in which tumour pieces were placed firstly for 2 h at 0°C and then for 4 h at 37°C. If, once again, the surviving fraction for immediate trypsinization is normalized to "S" then the values obtained were 9·6, 12·0 and 3·6 times "S" in 3 separate determinations. It therefore appears that the ability to increase surviving fraction may be "stored" at 0°C.

FIG. 3.—Change in surviving fraction of cells exposed *in vivo* to BLM for 30 min with length of subsequent holding *in vitro* before plating out. Circles refer to holding as tumour pieces. Triangles refer to holding as cell suspension. Solid symbols refer to holding at 37°C. Open symbols refer to holding at 0°C. Error bars indicate ±2 s.e. based on the total colony count on 4 replicate dishes.

*Inoculation of new tumours*

To ensure that the large difference in surviving fraction between cells cloned shortly after exposure to BLM and cells taken at later times is not an artefact of the cell culture procedure, we looked at the growth of tumours in mice given cells taken from treated animals. The growth of tumours in groups of 10 recipient mice are shown in Fig. 4. The solid lines are drawn through the mean tumour volumes of mice given 4 × $10^4$ or $10^3$ cells from untreated tumours at Day 0. The solid circles show the mean tumour volumes of mice given 4 × $10^4$ or $10^3$ cells from

FIG. 4.—Growth of tumours with time after inoculation. Solid lines are mean growth curves for inoculation of $4 \times 10^4$ and $10^3$ untreated cells. Closed circles, mean tumour volumes for inoculation of $4 \times 10^4$ and $10^3$ untreated cells and administration of BLM (10 mg/kg) to recipient mice 3 days later. Open circles, mean tumour volumes for inoculation of $4 \times 10^4$ cells taken from donor mice treated 24 h previously with BLM (10 mg/kg) Open squares, mean tumour volumes for inoculation of $4 \times 10^4$ cells taken from donor mice treated 1 h previously with BLM (10 mg/kg). Error bars represent $\pm$ 2 s.e. of the mean.

untreated tumours at Day 0 and then a single intraperitoneal injection of BLM (10 mg/kg) 3 days later. It may be seen that this treatment had little effect on the growth of the tumours for either size of inoculum. The open circles show the mean tumour volume for mice receiving $4 \times 10^4$ cells at Day 0 taken from mice whose tumours had been treated with BLM (10 mg/kg) 24 h previously. Again, there is no significant difference between these points and the points for an equal number of cells taken from untreated tumours. The open squares show the mean tumour volume for mice receiving $4 \times 10^4$ cells at Day 0 taken from mice whose tumours had been treated with BLM (10 mg/kg) one hour previously. In this case, the mean tumour volume is for the 4 tumour takes in a group of 10 recipients. Six recipients of this inoculum remained tumour-free for a period of 30 days and have been excluded from the mean. It is clear that in this case a very marked reduction in the ability of the cells to grow new tumours had occurred, thereby confirming the effect seen in the cell culture studies.

### In vitro *sensitivity of cells from tumours*

The survival of cells taken from solid tumours at 30 min after BLM was much lower than may have been expected from any of our *in vitro* data. We therefore attempted to establish a basis for comparison by treating in suspension, after trypinization, cells taken either from solid tumours or from late plateau phase cultures. The results are shown in Fig. 5. For late plateau phase cells the dose-response curve is little different whether the cells are treated on the flask surface before trypsinization or in sus-

FIG. 5.—Change in surviving fraction of cells with dose of BLM during 30 min drug exposure. Closed circles, late plateau phase cells *in vitro* treated as a monolayer before trypsinization. Open circles, late plateau phase cells *in vitro* treated in suspension after trypsinization. Squares, cells taken from tumours treated in suspension after trypsinization. Error bars indicate ±2 s.e. based on the total colony count in groups of 4 replicate plates.

pension immediately after trypsinization. If cells from solid tumours were treated in suspension for 30 min after trypsinization and resuspension, the surviving fraction was about 5% at a dose of 50–100 μg/ml. It is clear that even if there was a considerable concentration of drug into the tumour this result cannot explain the *in vivo* results of about 0·2% for a dose of 4 mg/kg either intraperitoneally or intravenously given 30 min before tumour excision.

### DISCUSSION

It is well established that the survival of mammalian cells following treatment with ionizing radiation may be modified by changing the post-irradiation conditions to which the cells are exposed. For instance, exposure to cyclohexamide (Phillips and Tolmach, 1966), exposure to low temperature (Whitmore and Gulyas, 1967) and incubation in a balanced salt solution (Belli and Shelton, 1969) have all been found to increase the surviving fraction if applied after irradiation. On the other hand, Phillips and Tolmach (1966) have found that post-irradiation treatment with inhibitors of DNA synthesis or exposure to 29°C cause a decrease in surviving fraction. The term "potentially lethal damage" has been used by Phillips and Tolmach (1966) to refer to damage which may be repaired under some circumstances but which is able to lead to cell death given certain post-irradiation conditions which by themselves have no effect on the survival of control cells.

Hahn and Little (1972) pointed out that conditions leading to increased survival following irradiation were those associated with a slowing down of the normal progression of cells through their life cycle. These workers then went on to examine the relative ability to repair radiation induced PLD of cells in the exponential and plateau phases of growth. In exponential phase, where the cells are almost all in a rapid state of proliferation, the radiation survival was the same whether the cells were subcultured immediately after irradiation or left in the original monolayer for several hours before subculture. On the other hand, crowded cultures in the plateau phase, where the amount of proliferation is greatly reduced, showed a considerable increase in surviving fraction if allowed to remain in the crowded state following irradiation than if immediately subcultured. Hahn and Little (1972) attributed this finding to the fact that cells in crowded cultures had more time to repair PLD before fixation of damage had occurred. The amount of PLD was greater at lower survival levels and this led to a reduction in the slope of the survival curve.

Subsequently, Little *et al.* (1973) were able to demonstrate a similar effect *in vivo* either in a crowded ascites tumour or a solid tumour in the mouse. The effect could not, however, be seen in the ascites tumour during exponential growth.

Repair of PLD following drug treatment has been investigated *in vitro* by

Ray *et al.* (1973). In this study repair of PLD occurred in both exponential and plateau phase cells folowing treatment with 5-FU, but only in plateau phase following treatment with bleomycin. Subsequently, however, using a different cell line, Barranco *et al.* (1975) showed repair of PLD following bleomycin treatment both in exponential and plateau phases. *In vivo*, Hahn *et al.* (1973) showed repair of PLD in the EMT6 mouse tumour following treatment with cyclophosphamide, 5-fluorouracil and bleomycin. We were able to confirm that BLM damage is repaired in solid EMT6 tumours over a wide range of sizes (Twentyman and Bleehen, 1974).

The studies described in the present paper add information on several aspects of this problem. We find that cells are able to repair PLD in exponential, early and late plateau phases, and to approximately the same extent from the same surviving fraction. Thus, the rate of proliferation of the treated cells does not, in our work, appear to be a crucial factor. Furthermore, the source of medium used during the subculture delay period does not seem to be an important factor, although our previous studies have shown (Twentyman *et al.*, 1975) that medium from plateau cultures is less able to support cell growth than exponential phase medium. This is in contrast to the finding by Little (1971) that conditioned medium can enhance the repair of PLD in irradiated cells. We have also shown that when cells are re-inoculated into flasks shortly before the experiment, PLD repair still occurs, thus ruling out local cell crowding as a factor. In addition, the possibility of an artefact due to temperature changes during subculture has been ruled out.

Our results *in vivo* show that the effect of PLD repair can be very much greater than has previously been described. Whereas Hahn *et al.* (1973) showed a recovery from about 10% to 70% survival between 2 and 24 h after relatively low BLM doses, we have found a recovery from about 0·2% to 90% between 30 min and 6 h, *i.e.* a factor of about 500 times. Again, we have ruled out temperature variations and the use of the enzyme, trypsin, during subculture as possible artefacts. We have also shown that PLD repair occurs in tumour pieces held *in vitro* for 4–6 h at 37°C after excision, does not occur at 0°C but may be stored at 0°C for later expression at 37°C.

It is extremely difficult to formulate any explanation which encompasses all the effects which we have observed. Having shown that both exponential and plateau phase cells repair PLD *in vitro*, we are left to discover what there is about the subculturing procedure which modifies survival, both *in vitro* and *in vivo*. There are several points which need to be made. Firstly, it has been shown by Barranco and Humphrey (1971) that, in addition to killing cells, BLM also induces a progression delay in the cell cycle. This delay could possibly allow repair of BLM lesions which would have been lethal if progression had continued unaltered. It would, however, be necessary to also show that the subculturing procedure was able to remove the progression delay, whilst medium change was not. We know of no evidence to support this. Studies by Schiaffini (unpublished data) have shown that repair of PLD following BLM treatment of Chinese hamster cells *in vitro* follows the same time course as rejoining of DNA strand breaks induced by BLM, with possible implications regarding the nature of the repair processes. Autoradiographic work by Fujimoto (1974) has shown that if $^{14}C$ labelled BLM is administered to mice bearing an ascites tumour, then the label is almost all at the cell surface at 2 h, has passed to the nuclear membrane by 4 h and visible necrosis sets in between 4 and 8 h. This may imply that BLM exerts a lethal effect on cells only if allowed a considerable period of time to enter the cell, having initially become absorbed on to the cell membrane. For this to explain the PLD story would require that some aspect of the subculturing procedure increased the rate of entry of BLM into the cell. Having ruled out the use of trypsin as the vital factor, at least *in vivo*, one is left with the possibility that any procedure, including mechanical disaggregation, is sufficiently traumatic that the membrane is damaged.

Another factor which requires explanation is the apparent discrepancy between the *in vivo* sensitivity if measured at 30 min and the *in vitro* sensitivity. One possible explanation is that the cell membrane is much more permeable to BLM when in the *in vivo* environment and that this permeability is lost when the tumour is made into a cell suspension and then exposed to the drug. It seems unlikely, however, that exposure to trypsin could cause this loss of permeability because the sensitivity of *in vitro* cells is similar when exposed to BLM either before or after trypsinization. An alternative explanation is that BLM is converted *in vivo* to a more active form. It does appear, however, that permeability of the cell membrane is a very important factor in determining the sensitivity of cells to BLM. It has been shown that the action of the antifungal polyene, pentamycin, which increases membrane permeability, can markedly increase the ability of BLM to inhibit DNA and RNA synthesis in cells (Nakashima *et al.*, 1974). Our own unpublished studies with pentamycin indicate that its use can increase by 100-fold the cell killing effect of BLM.

The balance of available evidence would therefore indicate that some aspect of the subculture procedure, as yet undetermined, either allows more BLM to enter the cell or else inhibits the repair of BLM damage. If the former explanation is the correct one, it may be that the phenomenon of PLD repair is really only an artefact induced by the procedure of making cell suspensions and has no significance with regard to cellular repair mechanisms. It would, however, leave open the possibility that manipulation of membrane permeability by other agents could be used to increase the efficacy of BLM. If, on the other hand, PLD repair does involve intracellular repair processes, then the possibility arises that BLM effectiveness could be increased by the combined use with agents known to act, in one way or another, as inhibitors of various types of repair. We are currently investigating these possibilities.

In this discussion, we have used the expression "repair of potentially lethal damage" in accordance with the usage which has become common over the last few years. At our present state of knowledge, however, it is in many ways an unfortunate expression in that it carries possible implications regarding mechanisms that are generally unjustified. In the absence of specific information regarding the nature of the damage sustained by cells, and evidence that repair of such damage is responsible for the increased survival with delayed trypsinization, great caution must be exercised. Furthermore, whilst increased survival with delayed trypsinization may be observed following exposure to both drugs and to x-rays, there is no necessity that the same mechanism be involved in both instances.

It is very clear from our results that in a situation where a tumour is treated *in vivo* and subsequently assayed *in vitro*, the result obtained can be extremely dependent upon the time after treatment at which the tumour is excised. It would appear therefore that any investigation of tumour response based on this type of assay should always include a careful investigation of the significance of this factor.

We thank Miss Stella Keller for her excellent technical assistance.

REFERENCES

Barranco, S. C. & Humphrey, R. M. (1971) The Effects of Bleomycin on Survival and Cell Pro-Progression in Chinese Hamster Cells *in vitro*. *Cancer Res.*, **31**, 128.

Barranco, S. C., Novak, J. K. & Humphrey, R. M. (1975) Studies on Recovery from Chemically Induced Damage in Mammalian Cells. *Cancer Res.*, **35**, 1194.

BELLI, J. A. & SHELTON, M. (1969) Potentially Lethal Radiation Damage: Repair by Mammalian Cells in Culture. *Science, N.Y.*, **165**, 490.

BLEEHEN, N. M., GILLIES, N. E. & TWENTYMAN, P. R. (1974) The Effect of Bleomycin and Radiation on Bacteria and Mammalian Cells in Culture. *Br. J. Radiol.*, **47**, 346.

FUJIMOTO, J. (1974) Radioautographic Studies on the Intracellular Distribution of Bleomycin C-14 in Mouse Tumor Cells. *Cancer Res.*, **34**, 2969.

HAHN, G. M. & LITTLE, J. B. (1972) Plateau Phase Cultures of Mammalian Cells: An *in vitro* Model for Human Cancer. *Cur. top. Radiat. Res.*, **8**, 39.

HAHN, G. M., RAY, G. R., GORDON, L. F. & KALLMAN R. F. (1973) Response of Solid Tumor Cells to Chemotherapeutic Agents *in vivo*. Cell Survival after 2 and 24 hour Exposure. *J. natn. Cancer Inst.*, **50**, 529.

LITTLE, J. B. (1971) Repair of Potentially Lethal Radiation Damage in Mammalian Cells: Enhancement by Conditional Medium from Stationary Cultures. *Int. J. Radiat. Biol.*, **20**, 87.

LITTLE, J. B., HAHN, G. M., FRINDEL, E. & TUBIANA, M. (1973) Repair of Potentially Lethal Radiation Damage *in vitro* and *in vivo*. *Radiology*, **106**, 689.

NAKASHIMA, T., KUWANO, M., MATSUI, K., KOMIYAMA, S., HIROTO, I. & ENDO, H. (1974) Potentiation of Bleomycin by an Antifungal Polyene, Pentamycin in Transformed Animal Cells. *Cancer Res.*, **34**, 3258.

PHILLIPS, R. A. & TOLMACH, L. J. (1966) Repair of Potentially Lethal Damage in X-irradiated HeLa Cells. *Radiat. Res.*, **29**, 413.

RAY, G. R., HAHN, G. M., BAGSHAW, M. A. & KURKJIAN, S. (1973) Cell Survival and Repair of Plateau Phase Cultures after Chemotherapy: Relevance to Tumour Therapy and to the *in vitro* Screening of New Agents. *Cancer Chemother. Rep.*, Pt 1, **57**, 473.

ROCKWELL, S. C., KALLMAN, R. F. & FAJARDO, L. F. (1972) Characteristics of a Serially Transplanted Mouse Mammary Tumor and its Tissue Culture Adapted Derivative. *J. natn. Cancer Inst.*, **49**, 735.

TAKABE, Y., WATANABE, M., MIYAMOTO, T. & TERASIMA, T. (1974) Demonstration of Repair of Potentially Lethal Damage in Plateau Phase Cells of Ehrlich Ascites Tumour after Exposure to Bleomycin. *Gann*, **65**, 559.

TWENTYMAN, P. R. & BLEEHEN, N. M. (1974) The Sensitivity to Bleomycin of a Solid Mouse Tumour at Different Stages of Growth. *Br. J. Cancer*, **30**, 469.

TWENTYMAN, P. R. & BLEEHEN, N. M. (1975) Changes in Sensitivity to Radiation and to Bleomycin Occurring During the Life History of Monolayer Cultures of a Mouse Tumour Cell Line. *Br. J. Cancer*, **31**, 68.

TWENTYMAN, P. R., WATSON, J. V., BLEEHEN, N. M. & ROWLES, P. M. (1975) Changes in Cell Proliferation Kinetics Occurring During the Life History of Monolayer Cultures of a Mouse Tumour Cell Line. *Cell tissue Kinet.*, **8**, 41.

WATSON, J. V. (1975) The Cell Proliferation Kinetics of the EMT6/M/AC Mouse Tumour at Four Volumes during Unperturbed Growth. *Cell Tissue Kinet.* In the press.

WHITMORE, G. F. & GULYAS, S. (1967) Studies on Recovery Processes in Mouse L Cells. *J. natn. Cancer Inst. Monog.*, **24**, 14.